# Supplementary figures and images for: Beta-caryophyllene enhances wound healing through multiple routes
Source: PLoS One. 2019 Dec 16;14(12):e0216104. doi: 10.1371/journal.pone.0216104 (PMC6913986; doi:10.1371/journal.pone.0216104)

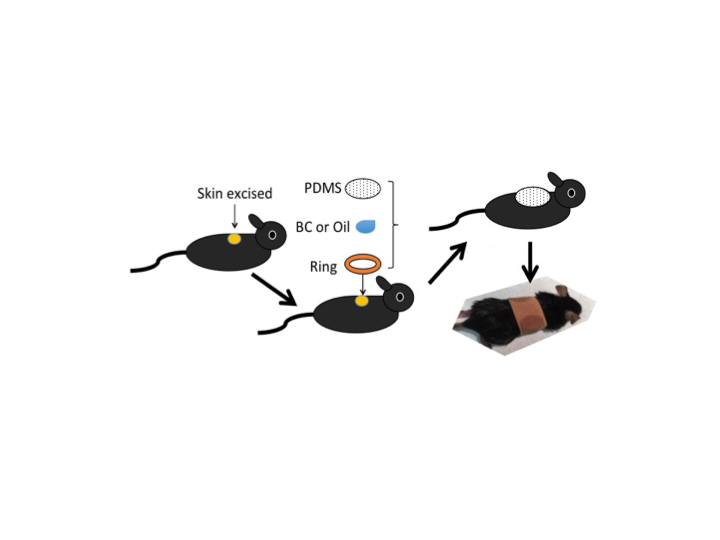

Supplement: S1 Fig — Small (5 mm x 5 mm) full-thickness excisions were made on the backs of adult mice and methods to hold the treatment buffer solution on the wounded area were developed. A silicone ring, which held 50 μL of buffer solution, was attached to the wounded area using a liquid sealing bandage (center). After the buffer solution was placed in the ring, a transparent lid made of polydimethylsiloxane (PDMS) was installed on top of the ring and was then sealed with the liquid sealing bandage (center and upper right). A gauze bandage was used to cover the body to prevent the mouse from removing the ring (lower right). (TIFF) [file pone.0216104.s001.tiff]

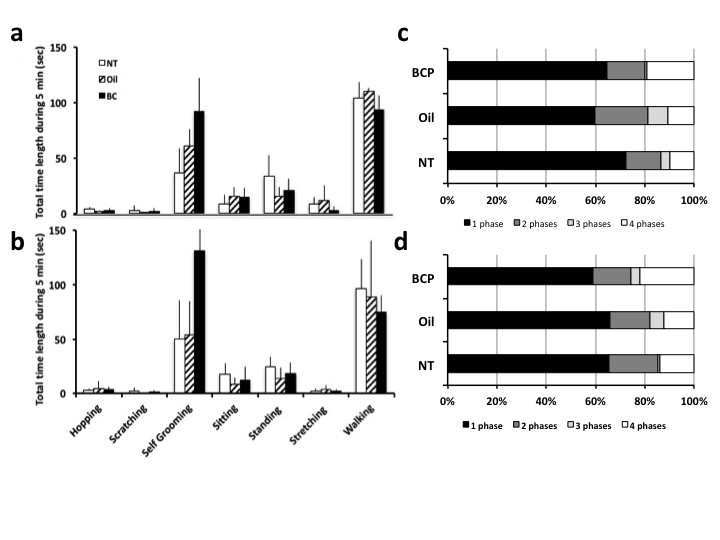

Supplement: S2 Fig — A concern in the use of BCP is that it may cause allergic responses. An oxidation product of BCP, i.e., caryophyllene oxide, is known as mild allergen [91]. To examine whether BCP has side effects, we conducted open-field tests on post-surgery day 1 (PS1) and 3 (PS3) and conducted behavioral analyses, specifically focusing on behavior patterns including scratching behaviors as an evidence of sensitization. In figures, NT group mice are mice without skin excision but went through all other steps and kept bandage on the same time length. There was almost no incidence of scratch behaviors on post-surgery day 1 (a) nor on post-surgery day 3 (b). Statistically significant differences were found in self-grooming behaviors of post-surgery day 3 (ANOVA, F2,15 = 4.585, P = 0.028). Self-grooming behaviors are known to increase at both high and low stress situations [92, 93]. If there are differences in the way BCP group mice did self-grooming behaviors, it could be due to BCP treatment. We classified self-grooming behaviors by the part in the body they groom and called them Phase 1 to Phase 4 following earlier studies [92, 93], and analyzed the self-grooming behaviors. We found no differences among the groups in the way self-grooming behaviors was conducted on both post-surgery day 1 and 3 (S2C and S2D Fig), which suggest that BCP treatment did not cause mice to self-groom in a different way. (c) and (d) show the % of short to long, full sequences of self-grooming behaviors depending on the group on post-surgery day 1 (c) (NT, n = 6, Oil, n = 6, BCP, n = 7) and 3 (d) (NT, n = 6, Oil n = 5, BCP, n = 7). Classification of self-grooming behavior is as follows [92, 93]: around the nose area (Phase I), around the face (Phase II), around the head and ears (Phase III), and to the body (Phase IV). Groomings toward the bandage were excluded from Phase IV to avoid the possibility that these grooming could be intention to remove bandages. Each incidence of grooming was classified into t [file pone.0216104.s002.tiff]

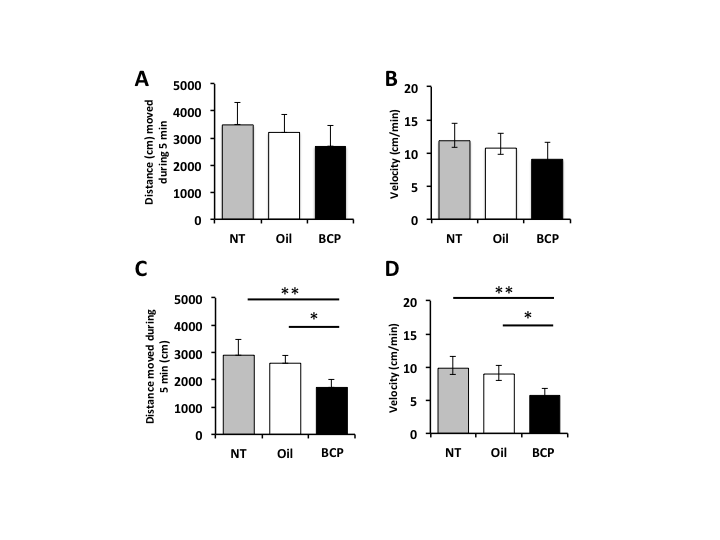

Supplement: S3 Fig — Open-field analyses of traveling distances and moving velocity revealed that on post-surgery day 1, there were no statistically significant differences among groups in the distance traveled (a) and velocity of movements (b) (ANOVA, distance, F2,21 = 2.205, P = 0.135; velocity, F2,21 = 2.647, P = 0.094; NT, n = 6, Oil, n = 6, BCP, n = 7). On post-surgery day 3, the distance traveled (c) was significantly shorter and the velocity was significantly slower (d) in the BCP group, whereas there were no differences between the Oil group and NT group (ANOVA, distance, F2,9 = 9.113, P = 0.007; velocity, F2,9 = 9.495, P = 0.006; NT, n = 6, Oil n = 5, BCP, n = 7). Linalool, a chemical compound included in lavender extracts, has anxiolytic effect in mice [68]. Whether the slower movements and increased self-grooming are signs that BCP has anxiolytic influence like linalool need to be addressed in future. Overall, these results showed that the impact of BCP on behavior was the longer time staying at a place doing self-grooming and the slow movements when the mice walked, which contain no signs of irritation from allergic responses. The BCP we used contains only 1.6% of caryophyllene oxide (S4 Fig, S1 Table) and fresh BCP was applied daily. The daily change may have contributed to reduce sensitization and allergic reactions. (TIFF) [file pone.0216104.s003.tiff]

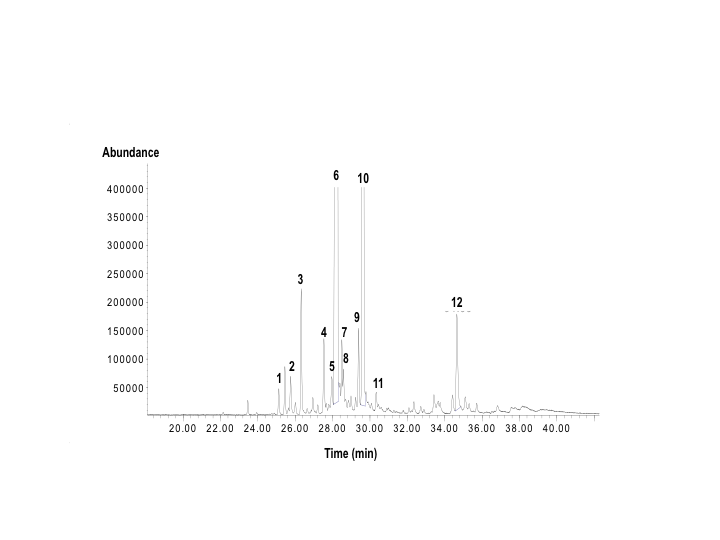

Supplement: S4 Fig — 1: cubebene, 2, 4, 5, 7, 8: sesquiterpenes of MW 204, 3: copaene, 6: BCP, 9: neoclovene, 10: α-caryophyllene, 11: 9-epi(E)-caryophyllene, 12: caryophyllene oxide. See S2 Table for details. (TIFF) [file pone.0216104.s004.tiff]

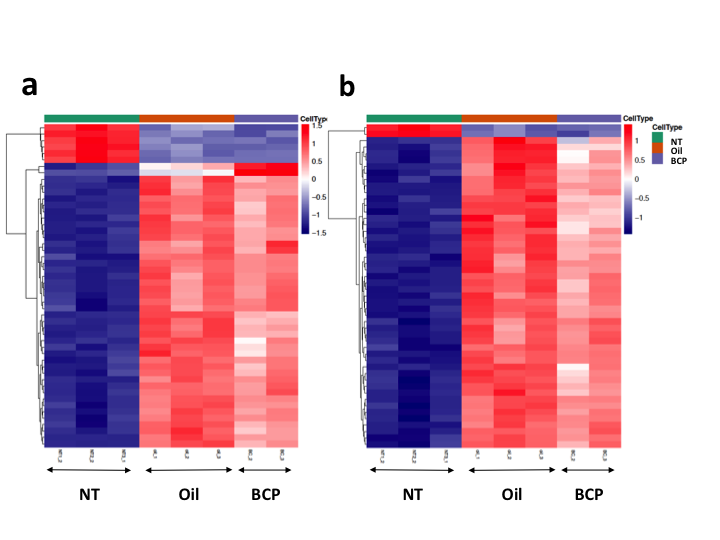

Supplement: S5 Fig — Results of RNA sequencing of post-surgery 17 hours skin and intact skin: Comparison between BCP and NT (a) and Oil and NT (b). Heatmap showing the top 50 significant gene expressions in the skin exposed to BCP (n = 2) or oil (n = 3), 17 to 18 hours post-surgery (inflammation stage), and in the skin of mice without skin excision (NT group) (n = 3). (TIF) [file pone.0216104.s005.tif]

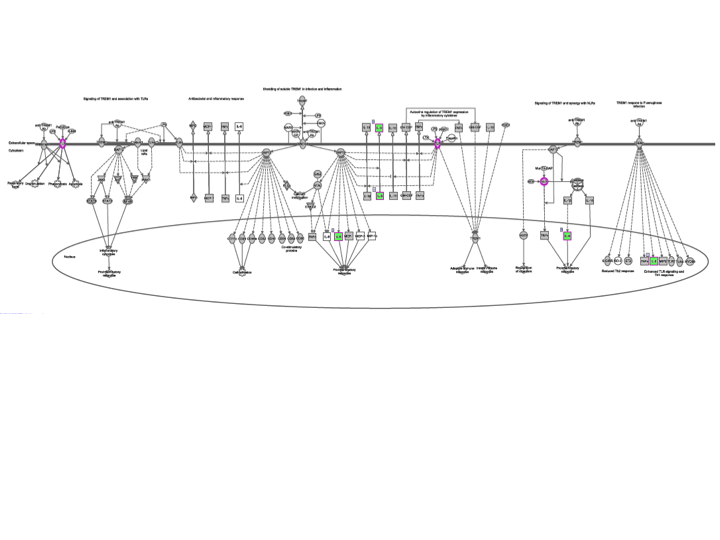

Supplement: S6 Fig — TREM1 signaling pathway showing the genes/groups of genes up-regulated (pink) and down-regulated (green) in BCP group compared to oil group. (TIFF) [file pone.0216104.s006.tiff]

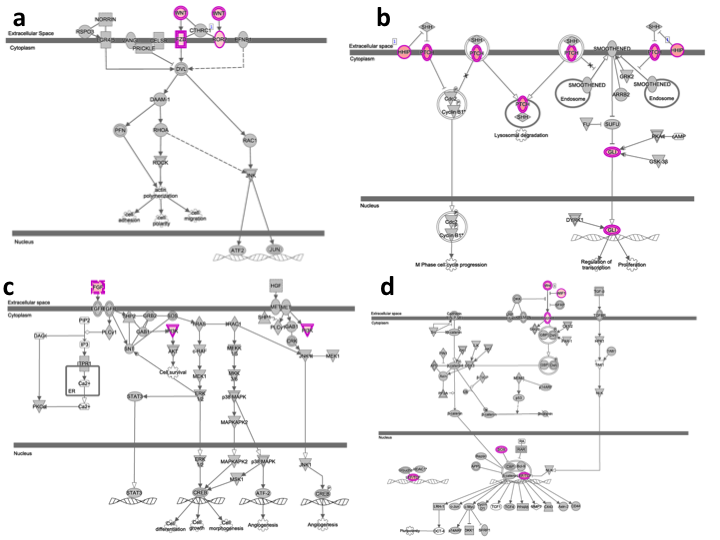

Supplement: S7 Fig — (a) Sonic hedgehog signaling (shh) pathway, (b) planar cell polarity (PCP) signaling pathway, (c) fibroblast growth factor (FGF) signaling pathway, and (d) Wnt beta-catenin signaling pathway. (TIFF) [file pone.0216104.s007.tiff]

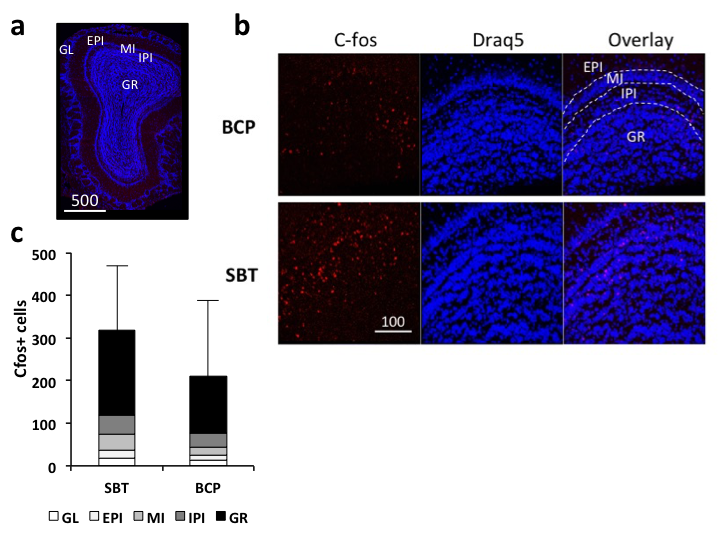

Supplement: S8 Fig — (a) Location where cfos was expressed profoundly. B (b) To test if mice can smell BCP, intact mice were exposed to BCP (n = 3) or male murine pheromone 2-sec-butyl-4,5-dihydrothiazole (SBT) (n = 5) [61, 62] for 60 min and perfused. Olfactory bulb was harvested, and processed to determine c-fos protein expression. Mice exposed to BCP and SBT both showed c-fos expression in the olfactory bulb. (c) Numbers of cfos+ cells were not statistically significantly different between the mice exposed to SBT and BCP because of the large variance in the expression of cfos. The expression was high in the females at estrous stage. Error bars indicates sd of total number of cells. GL: glomerular layer, EPI: external plexiform layer, MI: mitral cell layer, IPI: internal plexiform layer, GR: granule cell layer. (TIFF) [file pone.0216104.s008.tiff]

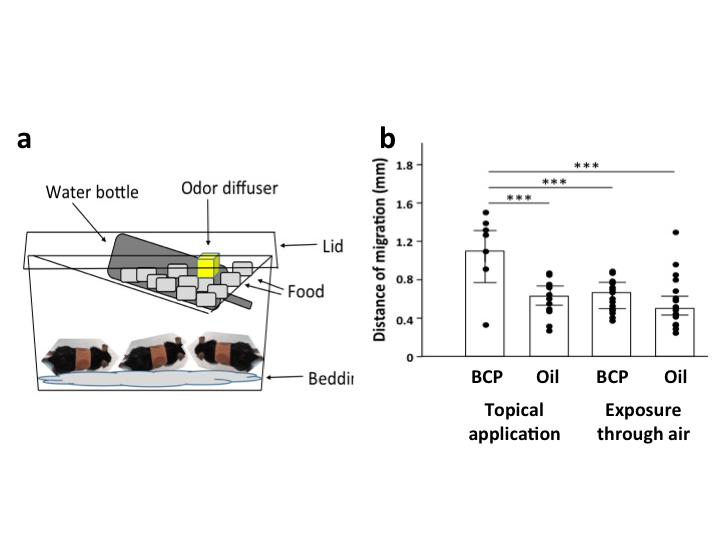

Supplement: S9 Fig — Mice were exposed to BCP or olive oil (Oil) through air by putting Q-tip soaked with either BCP or olive oil in a wire mesh and placing the wire mesh on top of the food provider (a). Food was placed as much as possible to avoid the possibility that mice can reach to the wire mesh. Mice went through surgery and surgery area was covered by the ring reservoir filled with olive oil and sealed by PDMS lid, which was covered by a bandage. (b) Exposure to BCP through air did not differ from control level and significantly different from topical application of BCP (ANOVA, BCP vs Oil F1,66 = 15.803, P<0.001, Topical vs Air F1,66 = 13.01, P = 0.001, Interaction F1,66 = 6.701, P = 0.012, BCP-Top, n = 16, Oil-Top, n = 16, BCP-Air, n = 18, Oil-Air, n = 20) ***: P<0.001, Tukey’s pairwise comparison was used for pair wise comparison. (TIFF) [file pone.0216104.s009.tiff]
