# Supplementary material for: Beta-caryophyllene enhances wound healing through multiple routes
Source: PLoS One. 2019 Dec 16;14(12):e0216104. doi: 10.1371/journal.pone.0216104 (PMC6913986; doi:10.1371/journal.pone.0216104)
Supplement: S1 Table — (PDF) [file pone.0216104.s010.pdf]

S1 Table. Beta-caryophyllene standard (W225207, Sigma-Aldrich) composition/GC-MS

No. in S4 Fig.	Rt (min)	Peak area x 10^6	Compound	%
1	25.11	0.831	Cubebene	0.1
2	25.75	1.379	a sesquiterpene, MW 204	0.2
3	26.31	5.299	Copaene	0.8
4	27.52	3.308	a sesquiterpene, MW 204	0.5
5	27.95	1.198	a sesquiterpene, MW 204	0.2
6	28.21	546.676	β-caryophyllene	81.8
7	28.49	1.704	a sesquiterpene, MW 204	0.3
8	28.59	0.4799	a sesquiterpene, MW 204	0.1
9	29.38	2.659	neoclovene	0.4
10	29.63	93.877	α-caryophyllene	14.0
11	20.33	0.681	9-epi(E)-caryophyllene	0.1
12	34.63	10.416	caryophyllene oxide	1.6
	SUM	668.508		100.0
