# Supplementary material for: Beta-caryophyllene enhances wound healing through multiple routes
Source: PLoS One. 2019 Dec 16;14(12):e0216104. doi: 10.1371/journal.pone.0216104 (PMC6913986; doi:10.1371/journal.pone.0216104)
Supplement: S2 Table — (PDF) [file pone.0216104.s011.pdf]

S2 Table. Olfactory receptor genes in the olfactory epithelium up-regulated after 1 hour exposure to BCP

Olfactory Receptor Gene	Log2FC	PValue
Olfr340	4.892	0.005
Olfr1480	1.881	0.028
Olfr103	1.656	0.001
Olfr1054	1.469	0.028
Olfr44	1.425	0.001
Olfr935	1.382	0.016
Olfr1152	1.325	0.041
Olfr616	1.246	0.037
Olfr1356	1.138	0.035
Olfr508	1.124	0.048
Olfr111	1.066	0.021
Olfr1258	1.013	0.002
Olfr33	0.928	0.042
Olfr874	0.880	0.026
Olfr599	0.810	0.022
Olfr92	0.780	0.046
Olfr716	0.736	0.015
Olfr701	0.729	0.048
Olfr1427	0.687	0.039
