# Supplementary material for: Beta-caryophyllene enhances wound healing through multiple routes
Source: PLoS One. 2019 Dec 16;14(12):e0216104. doi: 10.1371/journal.pone.0216104 (PMC6913986; doi:10.1371/journal.pone.0216104)
Supplement: S3 Table — (PDF) [file pone.0216104.s012.pdf]

S3 Table. Olfactory receptors expressed in skin after exposure to BCP, compared with Oil group

Olfactory receptor genes	Log2FC	Pvalue
Olfr267	2.575	0.355
Olfr624	1.336	0.544
Olfr912	0.497	0.661
Olfr1396	0.378	0.796
Olfr1034	0.344	0.859
Olfr110	0.256	0.780
Olfr212	0.211	0.925
Olfr482	0.075	0.737
Olfr558	-0.115	0.788
Olfr920	-0.14	0.777
Olfr1392	-0.151	0.965
Olfr750	-0.222	0.937
Olfr733	-0.243	0.929
Olfr78	-0.249	0.683
Olfr523	-0.279	0.862
Olfr560	-0.296	0.871
Olfr77	-0.466	0.668
Olfr346	-0.81	0.714
Olfr56	-0.836	0.264
Olfr466	-0.847	0.588
Olfr1378	-0.853	0.636
Olfr92	-0.883	0.604
Olfr1033	-0.898	0.221
Olfr111	-1.06	0.134
Olfr1383	-1.324	0.613
Olfr1388	-1.376	0.451
Olfr433	-1.806	0.405
Olfr1393	-2.218	0.395
Olfr432	-2.707	0.440
Olfr1564	-2.74	0.265
